# Concordance Between Estimated Fetal Weight by Ultrasound and Birth Weight and Its Association with Adverse Perinatal Outcomes

**DOI:** 10.3390/jcm14051757

**Published:** 2025-03-05

**Authors:** Cinara Carvalho Silva, Artur Bizinotto, Edward Araujo Júnior, Taciana Mara Rodrigues da Cunha Caldas, Alberto Borges Peixoto, Roberta Granese

**Affiliations:** 1 Gynecology and Obstetrics Service, Mario Palmério University Hospital—University of Uberaba (UNIUBE), Uberaba 38050-175, MG, Brazil; cinaracarvalho50@gmail.com (C.C.S.); arturbizinotto@outlook.com (A.B.); albertobpeixoto@gmail.com (A.B.P.); 2 Department of Obstetrics, Paulista School of Medicine, Federal University of São Paulo (EPM-UNIFESP), São Paulo 04023-062, SP, Brazil; araujojred@terra.com.br; 3 Discipline of Woman Health, Municipal University of São Caetano do Sul (USCS), São Caetano do Sul 09521-160, SP, Brazil; 4 Sabin Diagnostic Medicine, Uberaba 38010-160, MG, Brazil; taciana_c@yahoo.com.br; 5 Department of Obstetrics and Gynecology, Federal University of Triângulo Mineiro (UFTM), Uberaba 38025-440, MG, Brazil; 6 Department of Biomedical and Dental Sciences and Morphofunctional Imaging, “G. Martino” University Hospital, 98100 Messina, Italy

**Keywords:** pregnancy, fetus, biometry, ultrasound, estimated fetal weight, adverse perinatal outcomes

## Abstract

**Objective:** The aim of this study was to analyze the concordance between estimated fetal weight (EFW) and birth weight among ultrasound examinations with fetal biometry considered adequate and inadequate according to the International Society of Ultrasound in Obstetrics and Gynecology (ISUOG) guidelines, and its association with adverse perinatal outcomes. **Methods:** This was a retrospective and cross-sectional study carried out in two centers, involving parturients who delivered between 37 and 41 weeks. The following parameters were evaluated: biparietal (BPD), head circumference (HC), abdominal circumference (AC), and femur length (FL) measurement; EFW; the interval between the ultrasound and delivery; and the discrepancy between EFW and birth weight. A minimum of 140 participants were required to assess the association between EFW and birth weight. **Results:** A total of 305 ultrasound examinations were selected and divided into two groups: adequate (Group I n = 115) and inadequate (Group II n = 190) fetal biometry. The measurements of the cephalic pole (BPD + HC), AC, and FL were inadequate in 69.5% (132/190), 91.6% (175/190), and 72.1% (137/190) of participants, respectively. Group I had a lower gestational age at ultrasound examination (38.4 vs. 39.9 weeks, *p* < 0.001), a larger BPD measurement (93.9 vs. 91.6 mm, *p* = 0.001), a longer interval between ultrasound examination and delivery (3.8 vs. 2.0 days, *p* < 0.001), and a smaller discrepancy between EFW and birth weight (7.2 vs. 9.5%, *p* = 0.002) than Group II. In Group I, EFW was a strong significant predictor (AUC:0.94, 95%CI 0.85–0.99, *p* = 0.032) for identifying birth weight >4000 g. An EFW cut-off value of 4019.0 g was found to be a correct identifier for 85.7% of newborns with a birth weight >4000 g, with a false-positive rate of 13.7%. Group I had a lower risk of postpartum hemorrhage (7.0% vs. 15.8%, OR:0.39, 95%CI 0.17–0.90, *p* = 0.024) and composite adverse perinatal outcomes (13.0 vs. 23.3%, OR:0.49, 95%CI 0.26–0.94, *p* = 0.030) than Group II. In Group I patients, undergoing an ultrasound 7 days before delivery was an independent predictor of composite adverse perinatal outcomes [x^2^(1) = 4.9, OR:0.49, 95%CI: 0.26–0.94, R^2^ Nagelkerke:0.026, *p* = 0.030]. **Conclusions:** We observed a high rate of inadequate fetal biometry. There was poor concordance between EFW and birth weight. EFW was a strong significant predictor for identifying macrosomia. Ultrasound examination performed 7 days before delivery was an independent predictor of adverse perinatal outcomes.

## 1. Introduction

Over time, obstetrical ultrasound has become an important tool in fetal medicine, used to determine accurate gestational age and assess fetal growth and changes, among other applications [1,2]. The estimated fetal weight (EFW) is crucial for obstetric management, as it helps guide decisions about the mode of delivery and preparation for potential complications during birth. To assess fetal size, abdominal circumference (AC), femur length (FL), biparietal diameter (BPD), and head circumference (HC) are analyzed [1,2,3]. The use of multiple parameters has been shown to be more accurate than calculations using only one or two measurements [1,4], and the accuracy of estimated fetal weight (EFW) decreases as the birth weight increases [1]. For fetal size measurement to become an analysis of fetal growth, it is necessary to evaluate at least two biometric parameters at different times [2].

Epidemiologic, social, ethnic, maternal (height, weight, parity), and fetal (weight extremes, amniotic fluid volume, sex, presentation, placental thickness/position) parameters all interfere with the assessment of fetal growth [1,5]. Different populations have different patterns of intrauterine growth, and specific charts have been developed for these populations [1]. Gestational age at time of estimation also affects this parameter, which is more accurate between 25 and 30 weeks and between 37 and 41 weeks of gestation [4]. The mean fetal weight in the Brazilian population can be inferred from studies that analyze birth weight and fetal biometric parameters. According to a study carried out in the southern region of Brazil, the mean birth weight remained stable at 3200 g over 33 years [6]. Araujo Júnior et al. [7] established reference charts for fetal biometric parameters in 32,476 singleton pregnancies in Brazil and estimated the mean fetal weight to be 3169.5 g at around 38 weeks of gestation. Ferreira et al. [8], in a study of the Brazilian indigenous population, found the mean birth weight to be 3201 g.

Fetuses considered adequate for gestational age (AGA) present EFWs ranging from the 10th to the 90th percentile, with a margin of error of 7.5 to 22%, which is essentially due to two main factors: errors in obtaining the biometric measurements and errors in the mathematical formula used [2,4]. When Hadlock’s formula is used, the margin of error is reduced to 10% [4]. A quality ultrasound assessment requires, in addition to specially trained personnel, equipment with the minimum necessary conditions [3]. Even when using the specifications considered basic for ultrasound evaluation, studies indicate a variability of 10–15% in intra- and inter-observer analysis [4].

In order to standardize the parameters used in fetal biometry, studies have established technical criteria for obtaining measurements [3], with Salomon et al.’s criteria [2] being the most widely used. It is also interesting to standardize the formula for calculating EFW, with the Hadlook formula [9] with three parameters being superior to other formulas [10,11]. To ensure the accuracy of the technique and the quality of the tests performed, studies suggest determining the discrepancy between the EFW and birth weight, as well as performing periodic audits to correct factors that interfere with the ultrasound assessment [3,4]. According to one study, sonographers improved the quality of the images they sent after being made aware of the quality criteria and the number of measurements required [5].

The aim of this study was to analyze the concordance between EFW and birth weight among ultrasound examinations with fetal biometry considered adequate and inadequate according to the International Society of Ultrasound in Obstetrics and Gynecology (ISUOG) guidelines and its association with adverse perinatal outcomes.

## 2. Methods

This was a retrospective and cross-sectional study carried out in the Fetal Medicine Unit of the Department of Gynecology and Obstetrics of the University Hospital Mário Palmério and Sabin Diagnostic Medicine, Uberaba, Minas Gerais, Brazil, through an active search of the Astraia database (Astraia Software Gmbh^©^ 2000–2015, Munich, Germany) and electronic medical records using the SOUL MV system (MV Informática Nordeste Ltda. Recife, Brazil) in parturients who delivered between 37 and 41 weeks between March 2017 and November 2024. The study was approved by the Research Ethics Committee of the University of Uberaba (CAAE: 82203724.8.0000.5145).

This study included singleton, unselected, pregnancies between 37 and 40 weeks of gestation, calculated from the date of the last menstrual period and confirmed by first-trimester ultrasound, performed between 11 and 13 weeks and 6 days, without chromosomal anomalies, structural defects or genetic syndromes, which progressed to vaginal delivery or cesarean section and had an obstetric ultrasound examination performed at the service within 7 days prior to delivery. The ultrasound examinations of the study participants were randomly selected from the Astraia database and the Soul MV system’s image database during the study period

The following variables were evaluated to characterize the study population: maternal age, number of pregnancies, number of deliveries, alcohol consumption, smoking, use of illicit drugs, mode of delivery, birth weight [12], Apgar score at the 1st minute, and Apgar score at the 5th minute. To characterize the ultrasound examinations, the following variables were evaluated: type of device, gestational age at the time of ultrasound examination, BPD measurement, HC measurement, AC measurement, FL measurement, EFW [13], interval between ultrasound examination and delivery, and discrepancy between EFW and birth weight.

**Figure 1:**
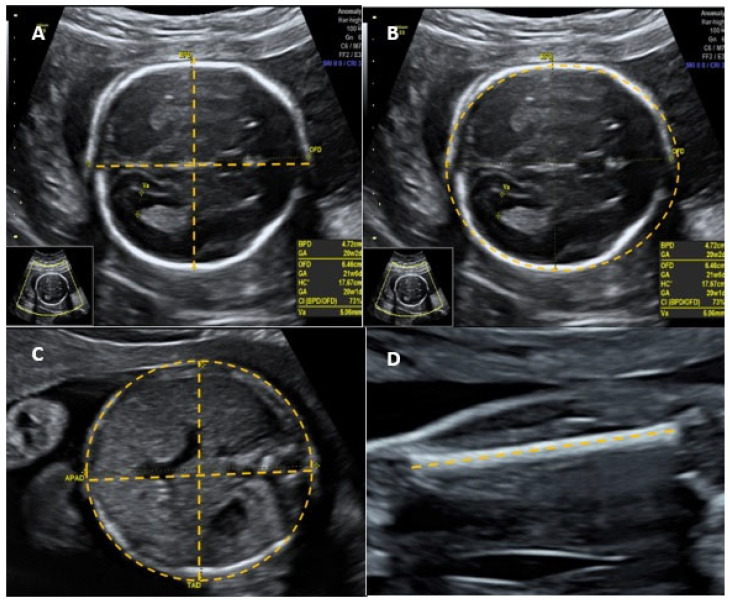
Measurements of biparietal diameter (BPD) (**A**), head circumference (HC) (**B**), abdominal circumference (AC) (**C**), and femur length (FL) (**D**) according to the International Society of Ultrasound in Obstetrics and Gynecology (ISUOG) guidelines.

The following parameters were considered adverse perinatal outcomes: Apgar score at the 1st minute < 7, Apgar score at the 5th minute < 7, neonatal intensive care unit (ICU) admission, maternal ICU admission, neonatal death in the first 48 h, postpartum hemorrhage, and maternal death. The presence of at least one adverse perinatal outcome was considered a composite adverse perinatal outcome. To consider the quality of the cephalic pole image as adequate, all of the following parameters had to be observed during BPD and HC measurements: plane between cerebral hemispheres symmetrical, thalamus visible, cavum of the septum pellucidum visible, cerebellum not visible, magnification of the fetal head occupying at least 50% of the screen, caliper correctly positioned on the outer edge of the anterior wall of the cephalic pole and on the outer edge of the posterior wall of the cephalic pole [3] (Figure 1A). To assess the HC, the calipers were positioned in the external–external position [2] (Figure 1B).

In order to consider the image quality of the fetal abdomen as adequate and to verify the AC measurement, all of the following parameters should be observed: the axial plane of the abdomen should be as rounded as possible while maintaining its symmetry; the stomach should be visible; the portal sinus should be visible; the kidneys should not be visible; magnification of the abdomen should occupy at least 50% of the screen; the calipers should be correctly positioned; the outer edge of the skin should be posterior to the spine and the outer edge of the skin of the anterior wall of the abdomen [2,3] (Figure 1C). For the image quality of the FL measurement to be considered adequate, all of the following parameters should be observed: there should be clear visualization of the end of the bone diaphysis, the angle between the sound beam and the femur should be between 45° and 90°; the magnification of the femur should occupy at least 50% of the screen; the calipers should be correctly positioned between the end of the two diaphyses [2,3] (Figure 1D).

To reduce interpretation bias in the quality of the plans for obtaining the sections used to determine fetal biometry, the principal investigator was first trained by analyzing 120 ultrasound examinations. Only they had achieved a kappa coefficient > 0.75 concordance with the fetal medicine specialist did data analysis begin. To account for the influence of ultrasound examination on the mode of delivery, we only included cases in which the indication for cesarean section was based solely on an EFW > 4000 g or an EFW < 10th percentile for gestational age according to the Hadlock table [14].

The GPower 3.1 program was used to calculate the sample size. Using an effect size of 0.25, a power of 80% with a 95% confidence interval (CI), and a significance level of 0.05, a minimum of 206 participants were needed to assess the association between EFW and mode of delivery. A minimum of 140 participants were required to assess the association between EFW and birth weight.

Data were collected in an Excel 2010 spreadsheet (Microsoft Corp., Redmond, WA, USA) and analyzed using SPSS statistical software version 20.0 (SPSS Inc., Chicago, IL, USA) and Prisma GraphPad version 7.0 (GraphPad Software, San Diego, CA, USA) and Stata version 18 (StataCorp LLC, College Station, TX, USA). D’Agostino’s and Pearson’s normality tests were used to analyze whether the values had a parametric distribution. Parametric distributed variables were presented as means and standard deviations. Non-parametrically distributed variables were presented as medians and minimum and maximum values. Student’s *t*-test (parametrically distributed variables) or the Mann–Whitney test (non-parametrically distributed variables) were used to compare variables between groups. The Chi-squared test was used to assess the association between the study group and the categorical variables. A general linear model was used to assess the effect of the study group on fetal biometric parameters, with gestational age at the ultrasound examination as a covariate.

Pearson’s correlation coefficient (r) was used to determine the correlation between EFW and birth weight, as well as the correlation between EFW and birth weight discrepancy. The Bland–Altman plot was used to assess the degree of concordance between EFW and birth weight. The concordance correlation coefficient (CCC) was used to calculate reliability and agreement using absolute and relative differences with their respective 95% CI. According to Martins and Nastri [15], agreement limits of relative differences > 50% indicate very poor concordance, those between 20% and 50% indicate poor concordance, those between 10% and 20% indicate moderate concordance, those between 5% and 10% indicate good concordance, and those <5% indicate very good concordance. The significance level for all tests was *p* < 0.05.

## 3. Results

**Figure 2:**
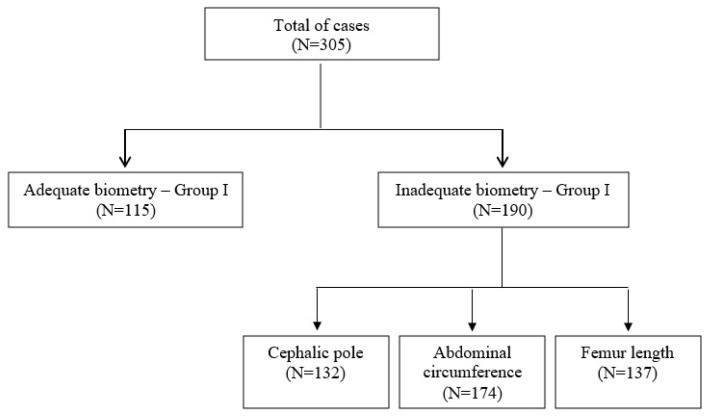
Flowchart of the included cases.

A total of 305 ultrasound examinations were selected and divided into two groups according to the standard of documentation of fetal biometric measurements. Ultrasound examinations with adequate fetal biometry according to ISUOG guidelines were assigned to Group I (N = 115) and ultrasound examinations with inadequate fetal biometry according to ISUOG guidelines were assigned to Group II (N = 190). Among the cases with inadequate fetal biometry, the assessment of the cephalic pole, AC, and FL was considered inadequate in 132, 174, and 137 cases, respectively (Figure 2).

The kappa coefficients for the cephalic pole, AC, and FL measurements were 0.85 (sensitivity: 93.9%/specificity: 91.7%, *p* < 0.0001), 0.75 (sensitivity: 94.1%/specificity: 88.5%, *p* < 0.001), and 0.89 (sensitivity: 100.0%/specificity: 94.7%, *p* < 0.001), respectively.

The measurement of the cephalic pole was inadequate in 69.5% (132/190) of the cases analyzed, the main reasons being as follows: thalamus not visible in 60.6% (80/132), incorrect positioning of the calipers in 60.6% (80/132), cavum of the septum pellucidum not identified in 55.3% (105/132), absence of symmetrical planes in 41.7% (55/132), insufficient image magnification in 30.3% (40/132), and identification of the cerebellum in 15.9% (21/132). The AC measurement was inadequate in 91.6% (175/190) of the cases analyzed, the main reasons being as follows: portal vein not visible in 83.9% (146/174), absence of stomach in 67.8% (118/174), incorrect positioning of calipers in 61.5% (107/174), absence of symmetrical planes in 42.5% (74/174), visible kidneys in 31.6% (55/174), and inadequate image magnification in 24.1% (42/174). FL measurements were inadequate in 72.1% (137/190) of the cases analyzed, with the main causes being as follows: inadequate image magnification in 84.7% (116/137), incorrect caliper positioning in 60.6% (83/137), extremities of bone not visible in 20.4% (28/137), and angle between sound beam and femur > 45° in 2.9% (4/137).

Ultrasound examinations with adequate fetal biometry (Group I) had a higher maternal age (28.0 vs. 25.0 years, *p* < 0.001) and lower gestational age at delivery (39.0 vs. 40.0 weeks, *p* < 0.001), as well as a higher prevalence of cesarean sections (63.5% vs. 47.9%, *p* = 0.005) than those with inadequate fetal biometry according to ISUOG guidelines (Group II) (Table 1).

Group I had a higher prevalence of ultrasound examinations performed with the GE Voluson E6 (97.4% vs. 54.2%, *p* < 0.001) and a lower prevalence of ultrasound examinations performed with the GE Logic P7 (2.6% vs. 45.3%) and Phillips Clear Vue 350 (0.0% vs. 0.5%, *p* < 0.001) than Group II. Group I had a lower gestational age at ultrasound examination (38.4 vs. 39.9 weeks, *p* < 0.001) than Group II. Group I had a larger BPD measurement (93.9 vs. 91.6 mm, *p* = 0.001) and a longer interval between ultrasound examination and delivery (3.8 vs. 2.0 days, *p* < 0.001) than Group II. Group I had a smaller discrepancy between EFW and birth weight (7.2 vs. 9.5%, *p* = 0.002) than Group II. Group I had a lower prevalence of EFW > 4000 g (4.3% vs. 17.9%, *p* < 0.001) and birth weight discrepancy > 15% (6.1% vs. 21.1%, *p* < 0.001) than Group II (Table 2).

Ultrasound examinations with an EFW > 4000 g were indicative of a higher risk of cesarean section (79.5% vs. 5.3%, OR: 69.8 95% CI: 27.1–180, *p* < 0.001) compared to that in patients whose ultrasound examinations showed an EFW < 4000 g (Table 3).

Ultrasound examinations with adequate fetal biometry and an EFW > 4000 g had a lower risk, higher sensitivity, and a higher positive predictive value for birth weight > 4000 g than those with inadequate fetal biometry (Table 4).

**Figure 3:**
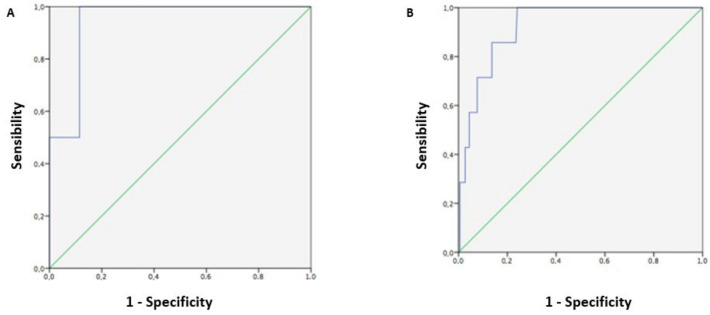
Receiver operating characteristics (ROC) curve for determining the best cut-off value of estimated fetal weight, performed 7 days before delivery, to predict birth weight > 4000 g, among ultrasound examinations with adequate (**A**) and inadequate (**B**) fetal biometry.

Among ultrasound examinations with adequate fetal biometry, EFW was a strong significant predictor (AUC: 0.94, 95% CI 0.85–0.99, *p* = 0.032) for identifying birth weight > 4000 g. An EFW cut-off value of 3779.5 g was able to correctly identify 100.0% of birth weight > 4000 g, with a false-positive rate of 11.5% (Figure 3A). Among ultrasound examinations with inadequate fetal biometry, EFW was a strong significant predictor (AUC: 0.92, 95% CI 0.86–0.98, *p* < 0.001) for identifying birth weight > 4000 g. An EFW cut-off value of 4019.0 g was able to correctly identify 85.7% of birth weights > 4000 g, with a false-positive rate of 13.7% (Figure 3B).

**Figure 4:**
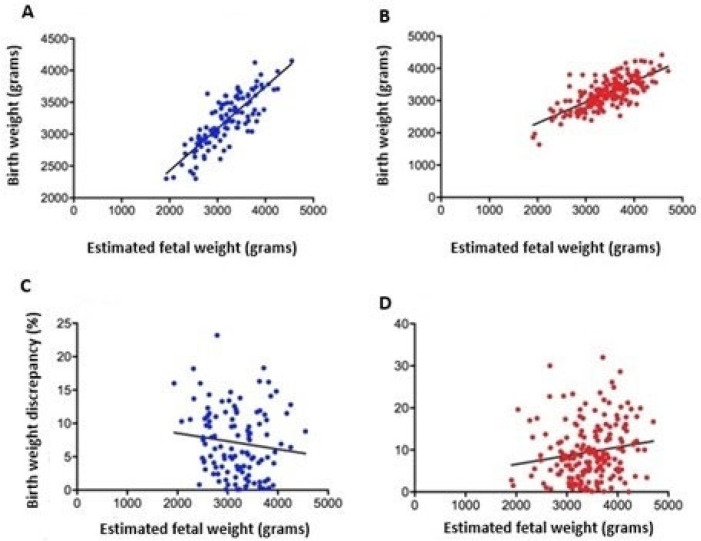
Correlation between estimated fetal weight and birth weight among ultrasound examinations with adequate (**A**) and inadequate (**B**) fetal biometry. Correlation between estimated fetal weight and birth weight discrepancy among ultrasound examinations with adequate (**C**) and inadequate (**D**) fetal biometry. Pearson correlation coefficient (r). *p* < 0.05.

A significant, positive, very strong (predictive) correlation was observed between EFW up to 7 days prior to delivery and birth weight (r = 0.82, *p* < 0.0001) in ultrasound examinations with adequate fetal biometry. It was found that 67.0% of birth weights were linearly related to EFW. A 1 g increase in EFW was responsible for a 0.6591 g increase in birth weight (Figure 4A). A significant, positive, strong correlation was observed between EFW up to 07 days prior to delivery and birth weight (r = 0.73, *p* < 0.0001) in ultrasound examinations with inadequate fetal biometry. It was found that 53.0% of birth weights were linearly related to EFW. A 1 g increase in EFW was responsible for an 0.6497 g increase in birth weight (Figure 4B).

A negative, non-significant correlation was observed between EFW performed up to 7 days prior to delivery and birth weight discrepancy (r = −0.12, *p* = 0.193) in ultrasound examinations with adequate fetal biometry (Figure 4C). A significant, very weak positive correlation was observed between EFW performed up to 7 days before delivery and birth weight discrepancy (r = 0.16, *p* = 0.024) in ultrasound examinations with inadequate fetal biometry. It was noted that 2.0% of the birth weight discrepancy was linearly related to EFW. A 1 g increase in EFW was responsible for increasing the birth weight discrepancy by 0.002% (Figure 4D).

There was concordance between EFW performed up to 7 days before delivery and birth weight (*p* = 0.361) in ultrasound examinations with adequate fetal biometry. There was poor concordance between EFW performed up to 7 days before delivery and birth weight [CCC = 0.80 (95%CI 0.73–0.86)]. The absolute mean difference between EFW and birth weight was 25 g lower. There was no concordance between fetal weight estimations made up to 7 days before delivery and birth weight (*p* < 0.001) in ultrasound examinations with inadequate fetal biometry. There was very poor concordance between fetal weight estimations made up to 7 days before delivery and birth weight [CCC = 0.66 (95%CI 0.59–0.73)]. The absolute mean difference between EFW and birth weight was 213.1 g higher (Table 5).

**Figure 5:**
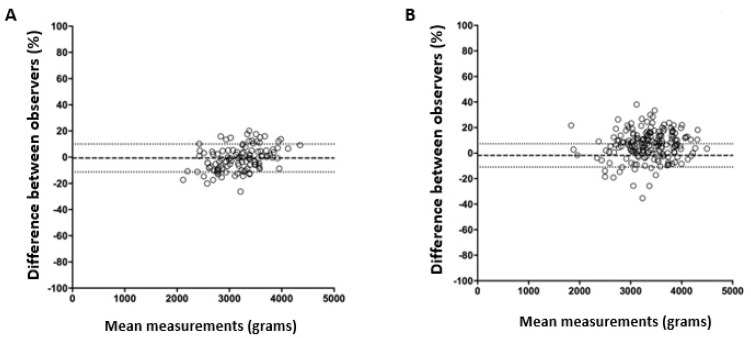
Bland–Altman plot for birth weight among ultrasound examinations with adequate (**A**) and inadequate (**B**) fetal biometry.

The mean relative difference between EFW and birth weight measurements was −1.3 in ultrasound examinations with adequate fetal biometry. The range of birth weights used to find 95% of the measurements was between −19.2 and 16.7 (Figure 5A). The mean relative difference between EFW and birth weight measurements was 6.2 in ultrasound examinations with inadequate fetal biometry. The expected birth weight range used to find 95% of the measurements was between −15.9 and 28.2 (Figure 5B).

Group I had a lower risk of postpartum hemorrhage (7.0% vs. 15.8%, OR: 0.39, 95% CI 0.17–0.90, *p* = 0.024) and composite adverse perinatal outcomes (13.0 vs. 23.3%, OR: 0.49, 95% CI 0.26–0.94, *p* = 0.030) than Group II (Table 6).

A forward stepwise binary logistic regression model was carried out using the adequacy of fetal biometry, number of previous pregnancies, EFW > 4000 g, and birth weight > 4000 g as independent variables, to assess the best model for predicting postpartum hemorrhage and composite adverse perinatal outcomes. Number of previous pregnancies (*p* = 0.081), EFW > 4000 g (*p* = 0.280) and birth weight > 4000 g (*p* = 0.622) were not predictors of postpartum hemorrhage. Thus, adequate fetal biometry performed 7 days before delivery was an independent predictor of postpartum hemorrhage [x^2^(2) = 8.3, OR: 0.39, 95% CI: 0.17–0.90, R^2^ Nagelkerke: 0.051, *p* = 0.024]. Number of previous pregnancies (*p* = 0.175), EFW > 4000 g (*p* = 0.139) and birth weight > 4000 g (*p* = 0.349) were not predictors of composite adverse perinatal outcomes. Thus, adequate fetal biometry performed 7 days before delivery was an independent predictor of composite adverse perinatal outcomes [x^2^(1) = 4.9, OR: 0.49, 95% CI: 0.26–0.94, R^2^ Nagelkerke: 0.026, *p* = 0.030].

## 4. Discussion

Obstetric ultrasound, including EFW measurement, has a direct impact on prenatal care, such as determining gestational age and mode of delivery. Understanding the margin of error in EFW calculations, as well as its causes and attempts to modify such errors, may result in fewer obstetric interventions and unnecessary procedures [4]. According to a study by Reboeiras et al. [4], the error in obtaining fetal biometric measurements is one of the factors that justifies the EFW margin of error, and is related to the mathematical formula used. Since all the ultrasound examinations in our study used Hadlock’s formula [9], which reduces the EFW error to 10%, we found that the main interference was the inadequate acquisition of images, especially of the AC.

As discussed in the study by Sánchez-Fernández et al. [5], there are some maternal and fetal variables that interfere with the accuracy of EFW, as well as the characteristics of the ultrasound device used [3,4]. In our study, ultrasound examinations with adequate fetal biometry had a higher prevalence in the GE Voluson E6 device, which has good image definition. In our study, an inexperienced examiner was initially trained by analyzing 120 ultrasound examinations, and only after they achieved a kappa coefficient of >0.75 concordance with the fetal medicine specialist did the data analysis begin. Combs et al. [16] developed a time-efficient, quantitative, objective, large-scale method to assess fetal biometric measurements for clinical practice. Fetal biometric measurements were converted to z-scores for standardization across gestational ages. The method provided an overview of the biometric measurements of all sonographers and physicians in a practice, allowing image audits to be focused on those whose measurements were outliers. Faschingbauer et al. [17] analyzed the influence of examiners and their experience on the quality of fetal biometric measurements. A retrospective study included 4607 fetal biometric examinations performed by 18 examiners at the beginning of their sonography training. The mean z scores for BPD, AC, and FL were statistically different from the expected value of 0; no significant differences were found for HC measurements. Regression analyses showed a significant effect of the number of ultrasound examinations on the distribution of z scores for each type of measurement. An increasing tendency toward either overestimation (HC) or underestimation (BPD, AC, and FL) was observed at the end of study.

Gestational age also affected the image quality, which is justified by the fact that the higher the gestational age, the greater the fetal insinuation, which increases the difficulty of obtaining biometric parameters, so that the accuracy of EFW decreases as the birth weight increases [1]. Rosen et al. [18] evaluated the accuracy of EFW in predicting birth weight and birth weight discordance in twin pregnancies. A retrospective cohort study included 2154 twin pregnancies and there was a strong correlation between EFW and birth weight for all twins. Advanced gestational age at ultrasound examination was correlated inversely with the mean absolute error of EFW. EFW accuracy was reduced for the non-presenting twin, the smaller cotwin, and when delivery occurred at an earlier gestational age.

EFW > 4000 g is associated with a significant increase in the likelihood of emergency cesarean section, postpartum hemorrhage, anal sphincter injuries, shoulder dystocia, and increased risk of hypoxic–ischemic encephalopathy [19,20,21]. In association with gestational diabetes, the risks associated with a birth weight ≥ 4000 g are even more pronounced, with a higher incidence of neonatal hypoglycemia, respiratory distress syndrome, and Erb’s palsy [21]. The EFW is crucial for obstetric management, as it helps guide decisions about the mode of delivery and preparation for potential complications during birth. Therefore, we believe that performing an ultrasound in accordance with appropriate technical recommendations is related to better concordance between EFW and birth weight, helping more reliably to manage the moment of delivery.

Among the indications for cesarean section, suspected fetal macrosomia stands out [22]. This association was confirmed in our study, which showed a higher risk of cesarean section in cases with an EFW > 4000 g. The shortest interval between ultrasound examination and delivery in the group with inadequate fetal biometry was found in the majority of cases with macrosomia and birth weight discrepancies >15%. In a systematic review, Coomarasamy et al. [10] determined the accuracy of EFW and AC in the prediction of macrosomia. There were 36 articles consisting of 63 accuracy studies (51 evaluating the accuracy of EFW, and 12 evaluating the accuracy of AC), including a total of 19,117 pregnant women. There were no significant differences between EFW and AC in predicting fetal macrosomia. In contrast with our results, Parikh et al. [23] observed that an EFW > 4000 g at < 38 weeks was associated with higher correlation between EFW and birth weight than ultrasound performed >38 weeks. The EFW to birth weight correlation was within 1.7% of the birth weight for cases in which the ultrasound performed at <38 weeks.

According to our study, ultrasound examinations with an adequate fetal biometry, in which an EFW > 4000 g was identified, showed a higher correlation with a birth weight > 4000 g. In this way, correct biometric assessment proved to be more efficient in predicting deviations from normal birth weight, thus avoiding unnecessary obstetric interventions. In our study, we used the Hadlock formula [9] to estimate fetal weight. Aye et al. [24] performed a prospective study of 170 consecutive pregnant women at term. The mean EFW using the Shepard formula was significantly higher than birth weight. The Shepard formula significantly overestimated macrosomia compared to birth weight. In a systematic review including 8530 pregnancies complicated by diabetes, Panunzi et al. [25] observed that the third-trimester ultrasound showed moderate accuracy in identifying fetuses with macrosomia with sensitivity of 71.2% and specificity of 88.6%. The interval between ultrasound examination and birth in two weeks showed the highest sensitivity and specificity (71.6% and 91.7%, respectively).

Our study showed poor and very poor concordance between EFW and birth weight in the adequate and inadequate fetal biometry, respectively. In contrast with our results, Souza et al. [26] evaluated the concordance, in relation to the 90th percentile, of ultrasound measurements of AC and EFW [World Health Organization (WHO) and the International Fetal and Newborn Growth Consortium for the 21st Century (Intergrowth-21st)] tables. Regarding birth weight, the best concordances were found for initial AC and with the final EFW in newborns of diabetic mothers.

This study showed that inadequate fetal biometry was an independent predictor of adverse perinatal outcomes such as postpartum hemorrhage. Kabiri et al. [27] compared the predictive performance of EFW, according to eight growth standards, to detect fetuses at risk for adverse perinatal outcomes. Fetuses with an EFW < 10th percentile or an EFW > 90th percentile were at increased risk of adverse perinatal outcomes according to all or some of the eight growth standards, respectively. They improved upon the detection of adverse perinatal outcomes by population-based (Intergrowth-21st) and customized (National Institute of Child Health and Human Development/Perinatology Research Branch) standards compared with the Hadlock and Fetal Medicine Foundation standards. Pretscher et al. [28] performed ultrasound examinations of 12,396 pregnant women within 7 days before delivery. Multivariate analyses demonstrated significant contributions for the prediction of obstetric intervention by HC and AC, whereas EFW did not reach significance. Multivariate analyses showed that significant contributions for the prediction of shoulder dystocia were provided only by AC. The overall detection rates for the prediction of adverse perinatal outcomes based on the different biometric parameters and EFWs were poor.

The study had limitations, such as being conducted in two small centers, the retrospective method of data collection, differences between practitioners during assessments, and possible co-morbidities in patients that could affect the data collection process.

## 5. Conclusions

We observed a high rate of inadequate fetal biometry in this study. EFW was found to be a strong significant predictor for identifying macrosomia. There was poor concordance between fetal weight estimations made up to 7 days before delivery and birth weight. An ultrasound examination performed 7 days before delivery was an independent predictor of adverse perinatal outcomes.

## Figures and Tables

**Table 1:** Clinical characteristics of the studied population.

Variable	Group I (N = 115)	Group II (N = 190)	*p*
Maternal age (years)	28.0 (18.0–46.0)	25.0 (18.0–41.0)	<0.001 ^∫^
Number of previous pregnancies	2.0 (1.0–8.0)	2.0 (1.0–7.0)	0.544 ^∫^
Number of previous deliveries	1.0 (0.0–5.0)	0.0 (0.0–6.0)	0.344 ^∫^
Alcohol consumption	0.9% (1/115)	0.0% (0/190)	0.198 ^∂^
Smoking	5.2% (6/115)	6.3% (12/190)	0.693 ^∂^
Illicit drug users	0.0% (0/115)	1.1% (2/190)	0.270 ^∂^
Gestational age at delivery (weeks)	39.0 (37.0–41.3)	40.0 (37.0–41.7)	<0.001 ^∫^
Mode of delivery			0.005 ^∂^
Vaginal	33.9% (39/115)	51.6% (98/190)	
Cesarean section	63.5% (73/115)	47.9% (91/190)	
Forceps	2.6% (3/115)	0.5% (1/190)	
Birth weight (grams)	3202 (410)	3255 (476)	0.329 ^†^
Birth weight > 4000 g	1.7% (2/115)	3.7% (7/190)	0.331 ^∂^
Birth weight < 10th percentile	11.3% (13/115)	17.4% (33/190)	0.152 ^∂^
Apgar score at the 1st minute	8.0 (4.0–10.0)	8.0 (0.0–9.0)	0.055 ^∫^
Apgar score at the 5th minute	9.0 (7.0–10.0)	9.0 (0.0–10.0)	0.188 ^∫^

Group I: adequate fetal biometry according to the ISUOG guidelines; Group II: inadequate fetal biometry according to the ISUOG guidelines. n = absolute number of cases; N = total number of cases. Mann–Whitney; ∫: median (minimum–maximum); Student’s *t*-test †: mean (standard deviation; Chi-squared; ∂: % (n/N). *p* < 0.05.

**Table 2:** Ultrasonographic characteristics of the studied population.

Variable	Group I (N = 115)	Group II (N = 190)	*p*
Ultrasound device			<0.001 ^∂^
GE Voluson E6	97.4% (112/115)	54.2% (103/190)	
GE Logic P7	2.6% (3/115)	45.3% (86/190)	
Phillips Clear Vue 350	0.0% (0/115)	0.5% (1/190)	
Gestational age at ultrasound examination (weeks)	38.4 (37.0–41.0)	39.9.0 (37.0–41.7)	<0.001 ^∫^
Biparietal diameter (mm)	93.9 (0.52)	91.6 (0.40)	0.001 ^√^
Head circumference (mm)	328.0 (1.83)	331.5 (1.40)	0.168 ^√^
Abdominal circumference (mm)	341.6 (2.83)	337.7 (2.16)	0.287 ^√^
Femur length (mm)	71.2 (5.55)	80.8 (4.24)	0.181 ^√^
Estimated fetal weight (grams)	3301.5 (44.8)	3393.0 (34.2)	0.117 ^√^
Estimated fetal weight > 4000 g	4.3% (5/115)	17.9% (34/190)	<0.001 ^∂^
Estimated fetal weight < 10th percentile	14.8% (17/115)	9.5% (18/190)	0.159 ^∂^
Ultrasound examination and delivery interval (days)	3.8 (0.22)	2.0 (0.17)	<0.001 ^√^
Discrepancy between estimated fetal weight and birth weight (%)	7.2 (0.59)	9.5 (0.45)	0.002 ^√^
Birth weight discrepancy > 15%	6.1% (7/115)	21.1% (40/190)	<0.001 ^∂^
Birth weight discrepancy > 10% and ≤ 15%	24.3% (28/115)	18.9% (36/190)	0.262 ^∂^
Birth weight discrepancy > 10%	30.4% (15/115)	39.5% (75/190)	0.111 ^∂^

Group I: adequate fetal biometry according to the ISUOG guidelines; Group II: inadequate fetal biometry according to the ISUOG guidelines. n = absolute number of cases; N = total number of cases Mann–Whitney ∫: median (minimum–maximum); Chi-squared ∂: % (n/N); general linear model √ (using gestational age at ultrasound examination as a covariate): mean (standard error). *p* < 0.05.

**Table 3:** Influence of estimated fetal weight 7 days before delivery on cesarean section rates.

Influence of Estimated Fetal Weight (EFW) on Cesarean Section Rates				
	EFW > 4000 g	EFW < 4000 g	OR (95% CI)	*p* *
Yes	79.5% (31/39)	5.3% (14/266)	69.8 (27.1–180)	<0.001
	EFW < 10th percentile	EFW > 10th percentile	OR (95% CI)	*p* *
Yes	8.6% (3/35)	11.5% (31/270)	0.72 (0.20–2.50)	0.607

OR: odds ratio; CI: confidence interval. Chi-squared *. *p* < 0.05.

**Table 4:** Odds ratio, sensitivity, specificity, positive predictive value and negative predictive value for birth weight > 4000 g, when estimated fetal weight > 4000 g 7 days before delivery.

	OR	95% CI	Sen	Spe	FPR	FNR	PPV	NPV	LH+	*p*
Appropriate fetal biometry	27.2	1.2–517.0	20.0%	99.1%	80.0%	0.9%	50.0%	96.5%	22.0	0.045
Inadequate fetal biometry	33.2	4.9–382.7	17.6%	99.4%	82.3	0.6%	85.7%	84.7%	27.5	<0.001

OR: odds ratio; CI: confidence interval; FPR: false-positive rate; FNR: false-negative rate; PPV: positive predictive value, NPV: negative predictive value, LH+: positive likelihood ratio; Sen: sensibility; Spe: specificity. Cross-reference table. *p* < 0.05.

**Table 5:** Concordance correlation coefficient values and their respective 95% confidence intervals in relation to reproducibility for cases with adequate and inadequate fetal biometry.

	CCC	MD	95% CI		Relative Difference			Absolute Difference		
					Bias	LoA		Bias	LoA	
Adequate fetal biometry										
Birth weight (grams)	0.80	−1.3	0.73	0.86	−1.3	−19.2	16.7	−25.0	−597.9	572.9
Inadequate fetal biometry										
Birth weight (grams)	0.66	6.2	0.59	0.73	6.2	−15.9	28.2	213.1	−522.7	735.8

CCC: concordance and correlation coefficient; MD: mean difference; CI: confidence interval; LoA: limit of agreement.

**Table 6:** Association between adequate fetal biometry and adverse perinatal outcomes.

Variable	Group I (N = 115)	Group II (N = 190)	OR (95% CI)	*p*
Apgar score at the 1st minute < 7	5.2% (6/115)	7.4% (14/190)	0.69 (0.25–1.85)	0.462
Apgar score at the 5th minute < 7	0.9% (1/115)	0.5% (1/190)	1.66 (0.10–26.8)	0.719
Neonatal ICU admission	1.7% (2/115)	3.2% (6/190)	0.54 (0.10–2.74)	0.452
Maternal ICU admission	0.0% (0/115)	1.1% (2/190)	0.32 (0.01–6.92)	0.272
Neonatal death in the first 48 h	0.0% (0/115)	0.0% (0/190)	*	*
Postpartum hemorrhage	7.0% (8/115)	15.8% (30/190)	0.39 (0.17–0.90)	0.024
Maternal death	0.0% (0/115)	0.0% (0/190)	*	*
Composite adverse perinatal outcomes	13.0% (15/115)	23.3% (44/190)	0.49 (0.26–0.94)	0.030

ICU: intensive care unit, n = absolute number of cases; N = total number of cases. OR: odds ratio calculated by binary logistic regression. * statistical analysis not performed % (n/N). *p* < 0.05.

## Data Availability

The data presented in this study are available on request from the corresponding author.
